# Electroacupuncture Improved the Function of Myocardial Ischemia Involved in the Hippocampus-Paraventricular Nucleus-Sympathetic Nerve Pathway

**DOI:** 10.1155/2018/2870676

**Published:** 2018-01-01

**Authors:** Shuai Cui, Yiping Zhou, Shengbing Wu, Jian Cao, Guoqi Zhu, Meiqi Zhou

**Affiliations:** ^1^Research Institute of Acupuncture and Meridian, Anhui University of Chinese Medicine, Hefei, China; ^2^Clinical Medical College of Acupuncture, Moxibustion and Rehabilitation, Guangzhou University of Chinese Medicine, Guangzhou, China; ^3^Key Laboratory of Xin'an Medicine, Ministry of Education, Anhui University of Chinese Medicine, Hefei, China; ^4^Department of Science and Technology, Anhui University of Chinese Medicine, Hefei, China

## Abstract

We investigated the hippocampus-paraventricular nucleus- (PVN-) sympathetic nerve pathway in electroacupuncture (EA) at the heart meridian for the treatment of myocardial ischemia by observing PVN neuronal discharge, sympathetic nerve discharge, and hemodynamics parameters. Sprague Dawley (SD) rats were equally divided into four groups: Sham, Model, Model + EA, and Model + EA + Lesion. The model rat was established by ligating the left anterior descending branch of the coronary artery. Changes in the sympathetic nerve discharge and hemodynamic parameters were observed. The Model + EA exhibited a significantly lower discharge frequency of PVN neurons compared with the Model. The Model + EA + Lesion had a significantly higher discharge frequency compared with the Model + EA. The total discharge frequency of PVN neurons and interneurons were positively correlated with the sympathetic nerve discharge. The total discharge frequency of PVN neurons was positively correlated with heart rate (HR) and negatively correlated with mean arterial pressure (MAP) and rate pressure product (RPP). The discharge frequency of interneurons was positively correlated with HR and negatively correlated with MAP and RPP. The hippocampus-PVN-sympathetic nerve pathway is involved in electroacupuncture at the heart meridian and interneurons are the key neurons in PVNs.

## 1. Introduction

Ischemic heart disease with clinical manifestation of acute myocardial ischemia (AMI) has high mortality rate (The Lancet, 2015). The current clinical understanding of ischemic heart disease has been formed from observing the heart and lacks overall observation and treatment of the nervous system. Some scholars [1] have suggested that the long-term effects of myocardial ischemia are closely related to the nervous system and that early intervention and protection of the nervous system contributes to the recovery of patients.

The pathway connecting the hippocampus and peripheral autonomic nervous system is involved in the regulation of the autonomic nervous system via the paraventricular nuclei (PVNs) of the hypothalamus and brain stem, nucleus tractus solitarius (NTS), and so on [2]. Our previous study confirmed that the improvement of myocardial ischemia by acupuncture at the heart meridian was associated with the sympathetic nervous system [3–5], and we found that AMI could cause the discharge of hippocampal CA1 neurons and changes in* c-fos* expression. Acupuncture at the heart meridian can reverse these changes, suggesting that the hippocampus is involved in acupuncture against myocardial ischemia [6]. However, the central mechanism remains unclear as to whether downstream PVNs regulating cardiovascular function are dominated by the hippocampus or not. To clarify the nerve fiber connection between the hippocampus and PVNs, we observed the effects of the hippocampus on the electrical activity of PVN neurons by the multichannel recording method. In addition, we noted the changes in sympathetic nerve discharge and hemodynamic parameters to investigate the central mechanisms of electroacupuncture at the heart meridian for the treatment of myocardial ischemia and the neural network for central cardiovascular regulation with the hippocampus as the important target. Thus, the present study provides a new idea for the treatment of ischemic heart disease.

## 2. Experimental Procedures

### 2.1. Animals

Forty clean male SD rats (250–300 g) were supplied by the feeding center at Anhui Medical University [number of animal license SCXK (Anhui) 2011-002]. Rats were housed in separate cages (Kangwei IR60) with an independent air supply system for 1 week at 22 ± 1°C and 60% relative humidity under natural light conditions, and food and water were supplied ad libitum. All animal procedures were conducted in accordance with the animal use guidelines of Anhui University of Chinese Medicine and Anhui Laboratory Animal Center.

### 2.2. Reagents and Instruments

The reagents used were as follows: chloral hydrate (Sinopharm Chemical Reagent Co., Ltd.); heparin sodium injection (Tianjin Biochemical Pharmaceutical Co., Ltd.); 0.9% sodium chloride injection (Anhui Fengyuan Pharmaceutical Co., Ltd.); kainic acid (KA, Sigma-Aldrich, St. Louis, MO).

The instruments used were as follows: 8-channel nickel alloy electrodes (diameter 35 *μ*m, Plexon Inc., HK); Plexon Multichannel Acquisition Processor (Plexon Inc., Dallas, TX, USA); BIOPAC multichannel physiological recorder (MP100, BIOPAC Systems Inc., USA); AcqKnowledge 4.1 for MP Systems (BIOPAC Systems Inc., USA); Offline Sorter (version 3.3.5, Plexon Inc., Dallas, TX, USA); NeuroExplorer (version 4.13, Nex Technologies, Lexington, MA, USA); multiarm electric brain stereotactic apparatus (Stoelting Co., Ltd., USA); Electronic Acupuncture Treatment Instrument (Huatuo brand SDZ-IV type, Suzhou Medical Products Co., Ltd., Suzhou, China); feedback-controlled DC electric heating pad (JR-1/2, Chengdu Taimeng Software Co., Ltd., Chengdu, China).

### 2.3. Animal Model and Treatment

The 40 healthy clean SD rats were equally and randomly divided into 4 groups: Sham group, Model group, Model + EA group, and Model + EA + Lesion group, 10 of each group. After some kind of operations, such as model replication, PVN damage, electrode array implantation, and carotid artery intubations, 24 rats have been survived, 8 rats died in model replication, 6 rats died in electrode array implantation operation, and 2 rats died in carotid artery intubations.

The AMI model was established by modifying the existing method [7]; the rats were not given tracheal intubation. After anesthesia with ether, a pericardial incision was performed to expose the heart through squeezing the chest, and the left anterior descending branch of the coronary artery (LAD) was ligated. The surgical procedure was completed within 2 minutes. The Sham group underwent the same surgical procedure without ligation of the LAD. High T wave and J point elevation ≥ 0.1 mV on electrocardiogram (ECG) indicated successful establishment of the AMI model.

The electrode array implantation has two steps: first, locating the PVN coordinate according to the rat brain map; second, using the dental cement to fix the electrode array.

The rat's neck operations contain three steps: first, isolating the artery and sympathetic nerve; second, carotid artery intubations; third, using the needle electrode to hook and record the sympathetic nerve.

The rat's nucleus damage (hippocampus CA1 region) adopts the method of chemical damage. All rats were anesthetized with 10% chloral hydrate (3.5 ml/kg) by intraperitoneal injection and were fixed on the brain stereotaxic apparatus in the prone position. Kainic acid (dose: 1 mg/ml) was injected into the hippocampus CA1 region bilaterally at the coordinates—Bregma—4.16 mm, LR 2.8 mm, and *H* 2.8–3.0 mm according to Paxions & Watson's rat brain atlas [8] (Figures S1A, S1B, S1C, and S1D). At three days after surgery, significant death of neurons was seen in the hippocampal CA1 region.

All above the experimental indexes (EEG, ECG, Sympathetic nerve, and hemodynamics) were recorded simultaneously (Figure 7(a)).

### 2.4. Meridian Selection and Electroacupuncture Parameters

The “Shenmen (HT7)-Tongli (HT5)” segment in the Shaoyin heart meridian of the hand was selected with reference to the human meridian line, the positioning criteria of acupuncture points in rats in* Chinese Veterinary Acupuncture*, and previous research results [3, 4]. Acupuncture procedure was as follows: for the rats in the Model + EA group and the Model + EA + Lesion group, three needles (Φ0.30 × 25 mm) were inserted at the “Shenmen (HT7)-Tongli (HT5)” segment with a spacing of 1 mm. A copper wire connecting the three needles in parallel as the “+” pole and another needle were inserted into the surrounding muscle as the “−” pole. Finally, “+” pole and “−” pole were connected to the Electronic Acupuncture Treatment Instrument. The EA parameter was set at two bursts of stimulation with a current of 1.1 mA at 2 Hz (duration: 30 min). The EA therapy was started at day 1 after surgery for consecutive three days in Model + EA group and Model + EA + Lesion group. Rats in Model group and Sham group deserved sham stimulations (with the instrument switch-off).

### 2.5. Peripheral Sympathetic Nerve Discharge Records

During recording, the room temperature was controlled at 26 ± 1°C, and the rat was placed on a heating pad maintained at 37 ± 2°C. The discharge frequency of the sympathetic nerve was recorded by the neural signal amplifier in the BIOPAC multichannel physiological recorder. The gain of the neural signal amplifier was adjusted to 500, the low-pass filter was adjusted to 100 Hz, and the high-pass filter was adjusted to 1 Hz. The two ends of the bipolar platinum electrode were connected to VIN + and VIN −, and the electrode tip was hooked on to the sympathetic nerve. The GND (ground) electrode was connected to the lower limb of the rat. The reference electrode was inserted into the subcutaneous tissue. The cotton pad soaked with liquid paraffin was placed under the nerve. After the electrode was hooked on the nerve, the anterior segment of the nerve was wrapped in the cotton pad to reduce noise and keep the nerve moist and warm. Acknowledge 3.8.1 software was used to record the neural signal and the threshold was set to ±0.04 V. After the records were saved, fast Fourier transformation (FFT) filtering was performed on the recorded signals offline, and the BAND STOP filtered out 50 Hz interference. The neural signals were recorded to determine the discharge frequency at 5 minutes after electroacupuncture.

### 2.6. Discharge Records of Neurons in Central Nucleus

All rats were anesthetized with 10% chloral hydrate (3.5 ml/kg) by intraperitoneal injection and then fixed on the brain stereotactic apparatus. The coordinates of PVN were set according to Paxions & Watson's rat brain atlas [8] as follows: Bregma −2.12 mm, LR 0.2–0.8 mm, and *H* 7.9–8.1 mm (Figures S1E and S1F). Craniotomy was performed to electrically move 8-channel microelectrode array to the target nucleus at 5 *μ*m/s. When the satisfactory discharge activity was observed, the stable neuronal discharge was recorded for 5 minutes. The Plexon multichannel acquisition system was used to record neuronal discharge (filter: 150–8000 Hz, sampling frequency: 40 kHz) and field potential (filter: 0.7–400 Hz, sampling frequency: 1 kHz). Using the Offline Sorter software, the neuronal discharge signals were screened according to the selection criteria (interspike interval (ISI): 1-2 ms, discharge rate < 0.5%). The selected neuronal discharge signals were subjected to the cluster analysis using manual and automatic methods. The pyramidal neurons and interneurons were distinguished based on the characteristics of neuronal discharge activity. The characteristics of pyramidal neurons were as follows: (1) low mean discharge frequency (0.5–10 Hz) and irregular discharge pattern; (2) the ISI histogram showed that the short ISI (3–10 ms) was dominant and exponential attenuation was present after 3–5 ms ISI; (3) wide waveform (>300 *μ*s). The characteristics of interneurons were as follows: (1) high mean discharge frequency (>5 Hz); (2) the ISI histogram presented delayed spikes and slower attenuation; (3) narrow waveform (<250 *μ*s) [9]. NeuroExplorer was used to analyze the waveform, frequency, characteristics of neuronal discharge, and correlation with field potential.

### 2.7. Hemodynamics and ECG Recording

All rats were anesthetized with 10% chloral hydrate (3.5 ml/kg) by intraperitoneal injection and fixed on the DC heating pad (maintained at 36-37°C) in the supine position. Using the conventional standard II lead ECG, the electrodes were inserted into the subcutaneous tissue of the limbs (right upper limb and left lower limb) in the rats. The BIOPAC multichannel physiological recorder was used to continuously monitor the ECG and the HR in the rats. Dr. Mao et al.'s method was used [10] to observe the respiratory, arterial blood pressure, and ECG waveform of the rats. After the rat achieved a stable status, the HR, MAP, and systolic blood pressure (SBP) were recorded and the RPP was calculated. The data were subjected to analysis using the computer built-in software, Acknowledge 4.1. The HR, MAP, and RPP values were compared between groups.

### 2.8. Statistical Methods

The statistical analysis was performed using SPSS 19.0 software (IBM SPSS, Inc.). Data were expressed as mean ± standard derivate. The difference between groups was analyzed by a one-way ANOVA. Homogeneity of the variance test was performed before the comparisons were made between groups. The LSD test was used for homogeneity of variance and Tamhane's* T*2 test for heterogeneity of variance.

Cluster analysis and autocorrelation analysis were used to distinguish and analyze the pattern of the PVN's neuron.

Real-time spectrum analysis was used to observe the all groups LFP's tendency of changes.

The PVN spike counts, interneuron, and pyramidal cell were correlated with the hemodynamic indexes (HR, MAP, and RPP) and sympathetic nerve discharge.

## 3. Results

### 3.1. The Hippocampus Involved in the Effect of the Heart Meridian Improve the Myocardial Ischemia by EA

**Figure 1:**
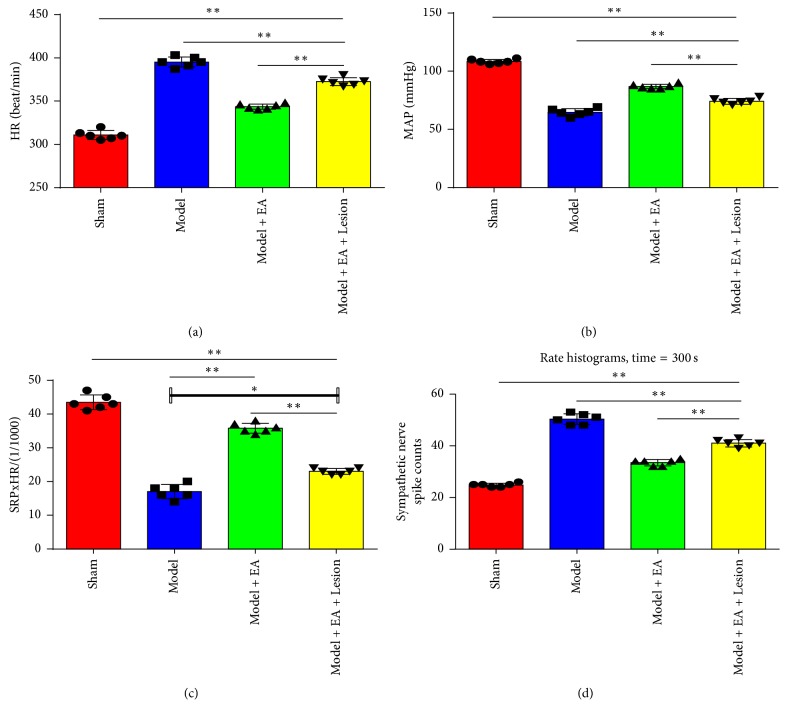
Comparisons of hemodynamic parameters and the sympathetic nerve discharge activity in each group. “(a)” is the HR histogram, “(b)” is the MAP histogram, “(c)” is the RPP histogram, and “(d)” is the discharge of sympathetic nerve histogram. Compared with the Sham group, the HR was significantly higher (*P* < 0.01); the MAP and RPP were significantly lower (*P* < 0.01); the discharge of sympathetic nerve was significantly higher (*P* < 0.01) in the Model group. Compared with the Model group, the HR was significantly lower (*P* < 0.01); the MAP was significantly higher (*P* < 0.01) in the Model + EA group and the Model + EA + Lesion group; the RPP was significantly higher (*P* < 0.01) in the Model + EA group and was higher (*P* < 0.05); the discharge of sympathetic nerve was significantly lower (*P* < 0.01) in the Model + EA + Lesion group. Compared with the Model + EA group, the HR was significantly higher (*P* < 0.01); the MAP and the RPP were significantly lower (*P* < 0.01); the discharge of sympathetic nerve was significantly higher (*P* < 0.01) in the Model + EA + Lesion group. The mean (*n* = 6), ^*∗*^ *P* < 0.05; ^*∗∗*^ *P* < 0.01.

HR, MAP, and RPP were recorded after three days of electroacupuncture. The Model group's HR was significantly higher (*P* < 0.01) than the other groups, the MAP and RPP were significantly lower than the other groups (*P* < 0.01). Compared with the Model group, the Model + EA group's HR demonstrated a significantly lower (*P* < 0.01), the MAP and RPP were significantly higher (*P* < 0.01 and *P* < 0.05, resp.). The Model + EA + Lesion group compared with the Model + EA group, the HR had a significantly higher (*P* < 0.01), and the MAP and RPP were significantly lower (*P* < 0.01) (Figures 1(a), 1(b), and 1(c)). These results suggest that the hippocampus is involved in electroacupuncture at the heart meridian for the treatment of myocardial ischemia.

### 3.2. The Hippocampi Involved in the Function of the Heart Meridian Adjust the Discharge of the Sympathetic Nerve by EA

Figure 1(d) shows that the stable discharge signal of the sympathetic nerve measured for 5 minutes was recorded immediately (0 min) after the last electroacupuncture for all experimental groups. The Model group showed a significantly higher discharge frequency of the sympathetic nerve compared with the Sham group (*P* < 0.01). Compared with the Model group, the Model + EA group displayed a significantly lower discharge frequency of the sympathetic nerve (*P* < 0.01). The Model + EA + Lesion group demonstrated a significantly higher discharge frequency of the sympathetic nerve compared with the Model + EA group (*P* < 0.01). These results suggest that the hippocampus is involved in electroacupuncture at the heart meridian for the treatment of myocardial ischemia by regulating the discharge activity of the sympathetic nerve.

### 3.3. The Hippocampus Involved in the Function of the Heart Meridian by EA Influences the Discharge of the PVN Neurons

**Figure 2:**
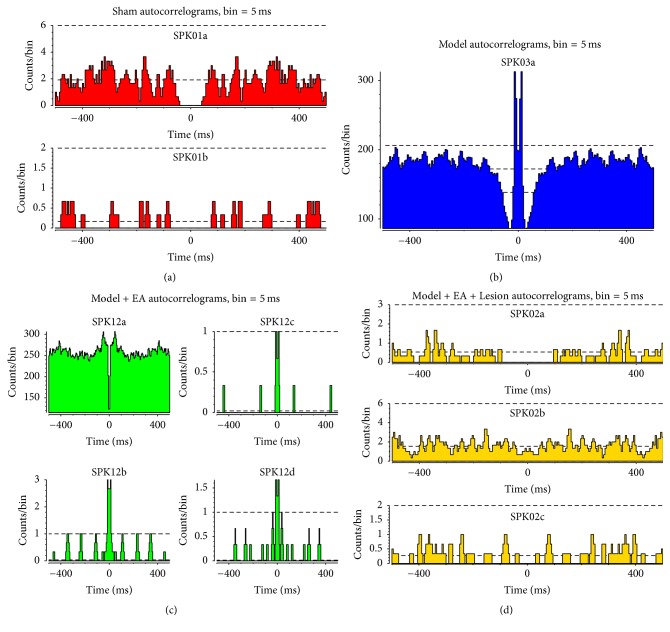
Distinguish the patterns of the PVN neurons by means of the autocorrelation analysis. “(a)” showed the discharge activity from the interneuron and pyramidal cell in the Sham group; “(b)” showed the discharge activity from the interneuron in the Model group; “(c)” showed the discharge activity from a single discharge pattern of interneuron and three discharge patterns of pyramidal cells in the Model + EA group; “(d)” showed the discharge activity from a single discharge pattern of interneuron and two discharge patterns of pyramidal cells in the Model + EA + Lesion group.

Multichannel* in vivo* recording techniques were used to record the stable discharge signals of the sympathetic nerves for 5 minutes immediately (0 min) after the last electroacupuncture in all 4 experimental groups. Figures S2A, S2B, S2C, and S2D showed that there were 2 PVN neurons with discharge in the Sham group, 1 in the Model group, 4 in the Model + EA group, and 3 in the Model + EA + Lesion group. The autocorrelation analysis was used to distinguish the discharge activities of the pyramidal cells and interneurons. Figures 2(a), 2(b), 2(c), and 2(d) showed that the Model group displayed a single discharge pattern of PVN neurons and only the discharge activity of interneurons was observed. The Model + EA group and the Model + EA + Lesion group showed discharge activity from the pyramidal cells and interneurons.

**Figure 3:**
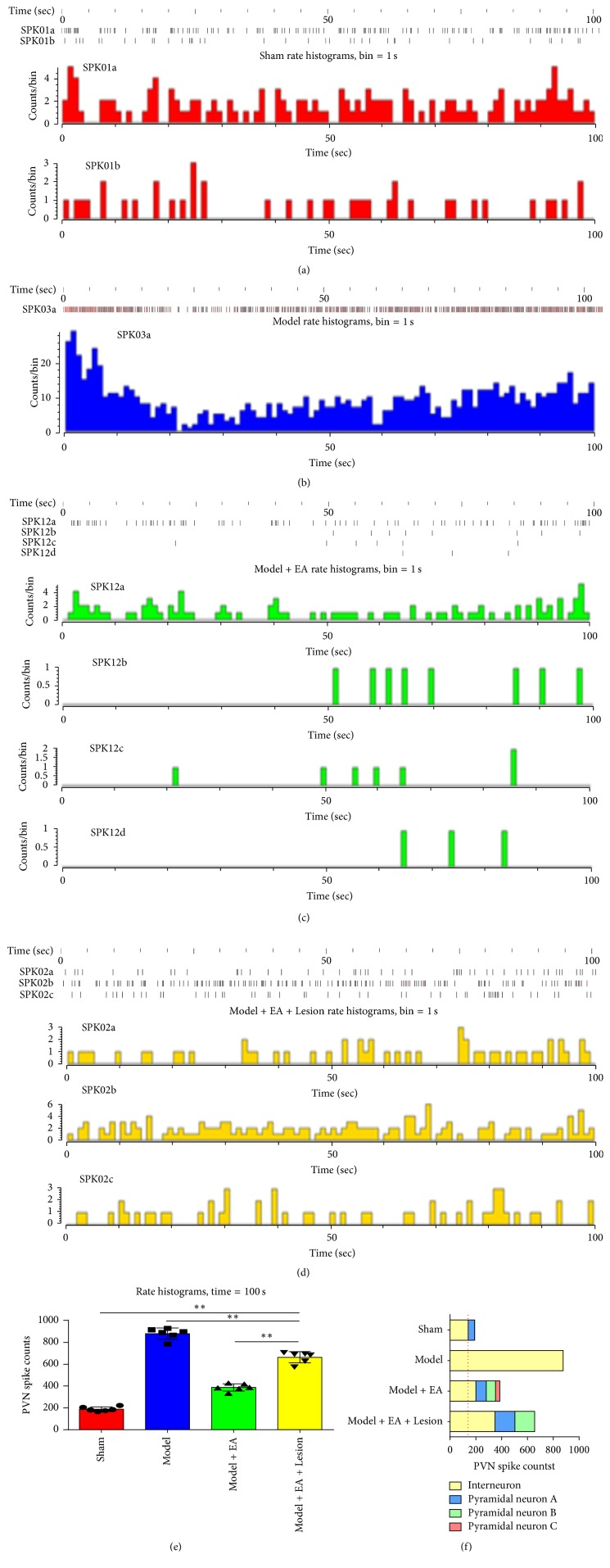
The discharge frequency and peak max of PVN and classification the neurons in each group. “(a),” “(b),” “(c),” and “(d)” showed that the frequency of PVN's neurons and max peak of PVN in each group with the time series (time = 100 s); “(e)” showed the comparisons of the discharge frequency of PVN neurons in each group. Compared the Sham group, the discharge frequency of PVN neurons was significantly higher (*P* < 0.01) in the Model group; compared the Model group, the discharge frequency of PVN neurons was significantly lower (*P* < 0.01) in the Model + EA group; compared the Model + EA group, the discharge frequency of PVN neurons was significantly higher (*P* < 0.01) in the Model + EA + Lesion group. The mean (*n* = 6), ^*∗∗*^ *P* < 0.01. “(f)” showed the classification of the interneurons and pyramidal cells between each groups.

Figures 3(a), 3(b), 3(c), and 3(d) showed that the time series (time = 100 s) of the neuronal discharges were converted to a histogram of discharge frequency. In the Sham group, the total discharge frequency of the PVN neurons was 190.6 ± 21.96 Hz and 50.2 ± 16.07 Hz for the pyramidal cells and 140.4 ± 9.07 Hz for the interneurons. In the Model group, the total discharge frequency of the PVN neurons was 877 ± 57.47 Hz, all of which were interneurons. In the Model + EA group, the total discharge frequency of the PVN neurons was 386.6 ± 37.1 Hz, 80.6 ± 4.78 Hz, 72 ± 3.94 Hz, and 34 ± 8.37 Hz for the pyramidal cells and 200 ± 21.48 Hz for the interneurons. In the Model + EA + Lesion group, the total discharge frequency of the PVN neurons was 656.4 ± 54.68 Hz, 154 ± 8.46 Hz, and 153.4 ± 13.13 Hz for the pyramidal cells and 349 ± 42.69 Hz for the interneurons.

The peak max of the pyramidal cells was 40 *μ*V (*n* = 3), and the peak max of the interneurons was 40 *μ*V (*n* = 10); in the Model group, the peak max of the pyramidal cells was 120 *μ*V (*n* = 29); in the Model + EA group, the peak max of the pyramidal cells was 30 *μ*V (*n* = 8), 70 *μ*V (*n* = 2), and 90 *μ*V (*n* = 3), and the peak max of the interneurons was 50 *μ*V (*n* = 5); in the Model + EA + Lesion group, the peak max of the pyramidal cells was 19 *μ*V (*n* = 6) and 5 *μ*V (*n* = 9), and the peak max of the interneurons was 28 *μ*V (*n* = 3).

Figures 3(e) and 3(f) showed the significant differences in the total discharge frequency of PVN neurons and the discharge frequency of the interneurons between groups (*P* < 0.01 and *P* < 0.01, resp.). These results suggest that the hippocampus is involved in electroacupuncture at the heart meridian for the treatment of myocardial ischemia by regulating the discharge activity of PVN neurons.

Real-time spectrum analysis was used to investigate the changes in spectral characteristics over time.  Figure S3 showed that, according to the intensity of the spectral energy of the local field potential (LFP), the 4 groups were sequenced as follows: the Model group > the Model + EA + Lesion group > the Model + EA group > the Sham group. These findings suggest that the hippocampus is involved in electroacupuncture at the heart meridian for the treatment of myocardial ischemia by regulating the spectral energy of the PVNs.

### 3.4. The PVN Neurons, Especially the Interneurons, Were Correlated with the Discharges of Sympathetic Nerve and Hemodynamics through the Heart Meridian by EA

**Figure 4:**
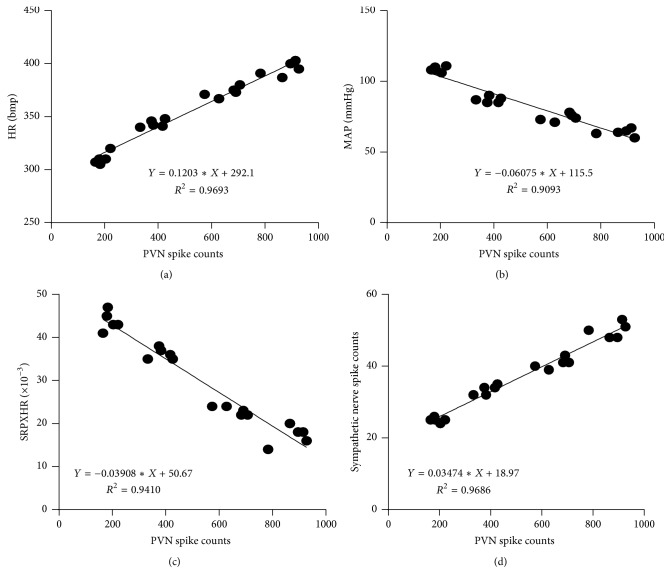
A correlation analysis was performed between the total discharge frequency of the PVN neurons and the total discharge frequency of the sympathetic nerve and hemodynamic parameters. “(a)” is correlated with the HR (*P* < 0.01, *r* = 0.9845); “(b)” is correlated with the MAP (*P* < 0.01, *r* = −0.9536); “(c)” is correlated with the RPP (*P* < 0.01, *r* = −0.9701); “(d)” is correlated with the discharge frequency of the sympathetic nerve (*P* < 0.01, *r* = 0.9842).

**Figure 5:**
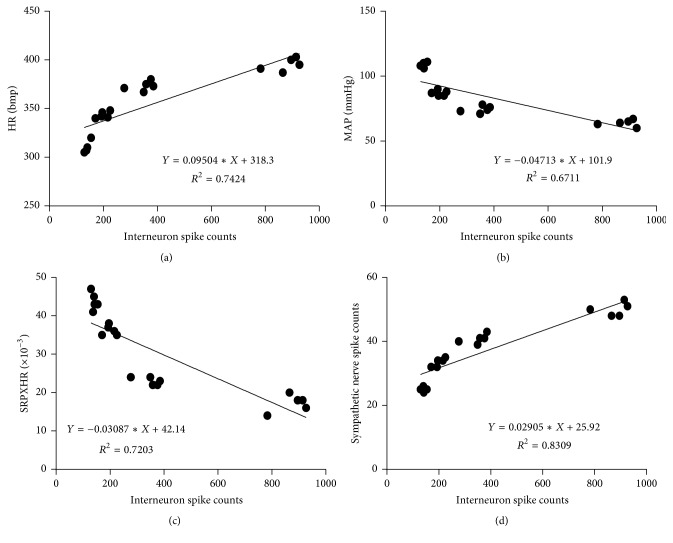
A correlation analysis was performed between the discharge frequency of the PVN interneurons and the total discharge frequency of the sympathetic nerve and hemodynamic parameters. “(a)” is correlated with the HR (*P* < 0.01, *r* = 0.8616); “(b)” is correlated with the MAP (*P* < 0.01, *r* = −0.8192); “(c)” is correlated with the RPP (*P* < 0.01, *r* = −0.8487); “(d)” is correlated with the discharge frequency of the sympathetic nerve (*P* < 0.01, *r* = 0.9115).

**Figure 6:**
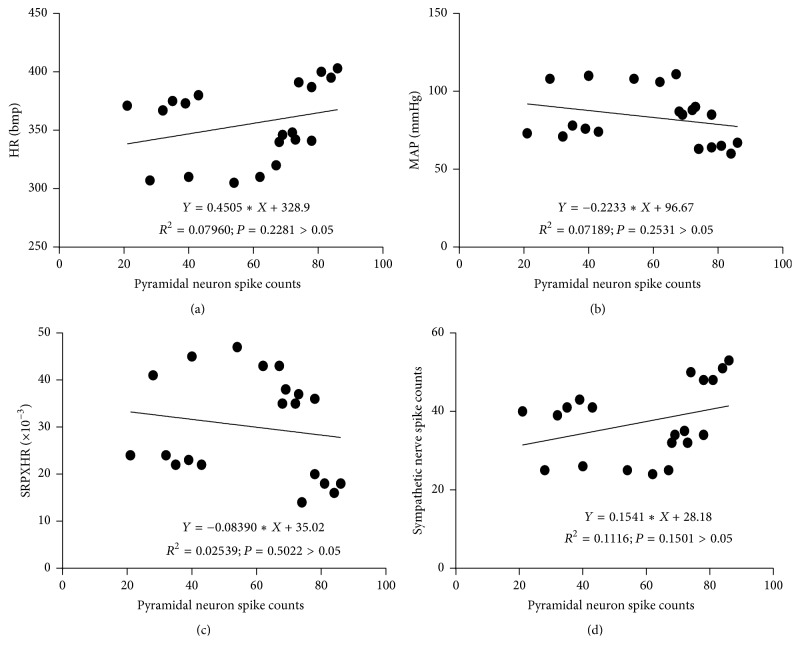
A correlation analysis was performed between the discharge frequency of the pyramidal cells in the PVNs and the total discharge frequency of the sympathetic nerve and hemodynamic parameters. “(a)” is correlated with the HR (*P* > 0.05, *r* = −0.2821); “(b)” is correlated with the MAP (*P* > 0.05, *r* = −0.2681); “(c)” is correlated with the RPP (*P* > 0.05, *r* = −0.1593); “(d)” is correlated with the discharge frequency of the sympathetic nerve (*P* > 0.05, *r* = 0.3341).

A correlation analysis was performed between the total discharge frequency of the PVN neurons and the total discharge frequency of the sympathetic nerve and hemodynamic parameters. Figures 4(d) and 5(d) showed that the total discharge frequency of the PVN neurons and the discharge frequency of the PVN interneurons were positively correlated with the discharge frequency of the sympathetic nerve (*P* < 0.01, *r* = 0.9842 and *P* < 0.01, *r* = 0.9115, resp.). However, the discharge frequency of the pyramidal cells in the PVNs was not correlated with the discharge frequency of the sympathetic nerve (*P* > 0.05, *r* = 0.3341) (Figure 6(d)). The total discharge frequency of the PVN neurons was positively correlated with HR (*P* < 0.01, *r* = 0.9845) and negatively correlated with MAP and RPP (*P* < 0.01, *r* = −0.9536 and *P* < 0.01, *r* = −0.9701, resp.) (Figures 4(a), 4(b), and 4(c)). The discharge frequency of the PVN interneurons was positively correlated with HR (*P* < 0.01, *r* = 0.8616) and negatively correlated with MAP and RPP (*P* < 0.01, *r* = −0.8192 and *P* < 0.01, *r* = −0.8487, resp.) (Figures 5(a), 5(b), and 5(c)), whereas the discharge frequency of the pyramidal cells in PVNs was not correlated with HR, MAP, or RPP (*P* > 0.05, *r* = −0.2821; *P* > 0.05, *r* = −0.2681; and *P* > 0.05, *r* = −0.1593, resp.) (Figures 6(a), 6(b), and 6(c)). These results suggest that the total discharge frequency of the PVN neurons and the discharge frequency of the PVN interneurons are correlated with the discharge frequency of the sympathetic nerve and hemodynamic parameters.

## 4. Discussion

The association between the meridian-viscera and the brain is the integration and breakthrough point for Chinese and Western medicine theories [11]. The brain has a direct or indirect connection with meridians to achieve the regulation of the viscera, limbs, and bones. It is a correct and feasible direction and an important current trend to investigate the association between the meridian-viscera and the brain from the angle of the limbic system-hypothalamus-autonomic nervous system [12].

Acupuncture has multitarget, multilevel, and multichannel characteristics to improve myocardial ischemia. A large number of experimental studies have shown that acupuncture can improve myocardial ischemia through a variety of mechanisms [13, 14]. Acupuncture results in the protection against myocardial ischemia due to the regulation of lipid peroxidation, myocardial energy metabolism, myocardial enzymes, and ion channels as well as the influence on ultrastructure and apoptosis. In terms of its central regulatory mechanism, acupuncture protects against myocardial ischemia through the inhibition of the cardiac sympathetic nervous system (opioid-PKC-dependent pathway) [15].

The hippocampus is one of the limbic forebrain structures. The hippocampus is not only an important center for regulating memory and cognition but also an important center for regulating cardiovascular function. A large number of studies have shown that the hippocampus can inhibit HPA axis activity [16]. Electrical stimulation of the hippocampus can inhibit stress-induced corticosteroid secretion; however, damage of the hippocampus or dorsal side of the hippocampus can increase CRHmRNA and AVPmRNA in the hypothalamic PVN [17]. By affecting the activities of cardiovascular neurons in the hypothalamus, brain stem, and other parts, the hippocampus can promote or inhibit the activities of the primary center to cause the cardiovascular activity to adapt to a variety of behaviors of the body. Previous studies [18, 19] found that myocardial ischemia can cause progressive loss of cerebral perfusion, especially for the brain areas, which are sensitive to ischemic injury, such as the hippocampus, and can induce the apoptosis of neurons in the limbic system. In addition, studies have found that prolonged electroacupuncture can regulate the interrelationships of the internal functions of the whole-brain network [20]. Furthermore, through electroacupuncture stimulation, it has been shown that the limbic/paralimbic regions, such as the amygdala, hippocampus, and anterior cingulate cortex, form a neural network center [20]. During the process of acupuncture, these brain regions play an important role in the regulation of the specific functions of the whole-brain network [21].

PVN is the central integrated area and the most important central site, which directly regulates sympathetic efferent activity. PVN is involved in the regulation of stress, endocrine function, and visceral function (gastrointestinal, renal, and cardiovascular activities) [11, 22] and is one of the important central structures, which regulates sympathetic nerve activity and arterial blood pressure [23, 24]. Chen et al. [25] showed that after the hypothalamic PVN of a rabbit is subjected to electrical damage, the protective effect of electroacupuncture on ischemic myocardium is significantly reduced, suggesting that the PVN is involved in the protective effect of electroacupuncture against myocardial ischemia.

In recent years, the multichannel* in vivo* recording technique has been widely used in neuroscience research because of its real-time performance and the advantage of recording many neurons [26–29]. The time series (spike) of a single neuron discharge and the signals recorded using the multichannel* in vivo* recording technique belong to the time-domain signal, wherein LFP is the continuous time-domain signal and spike is the discrete time-domain signal. These two types of signals are transformed into frequency-domain signals by spectral analysis to investigate the changes in frequency in each experimental group. Recorded single neurons are broadly divided into two categories, including pyramidal cells and interneurons. The pyramidal cells exhibit an obvious characteristic of a cluster discharge, whereas some interneurons show this characteristic. The typical interneuron discharge presents multiple equidistant peaks, suggesting that the discharge has an obvious characteristic of a periodic distribution.

**Figure 7:**
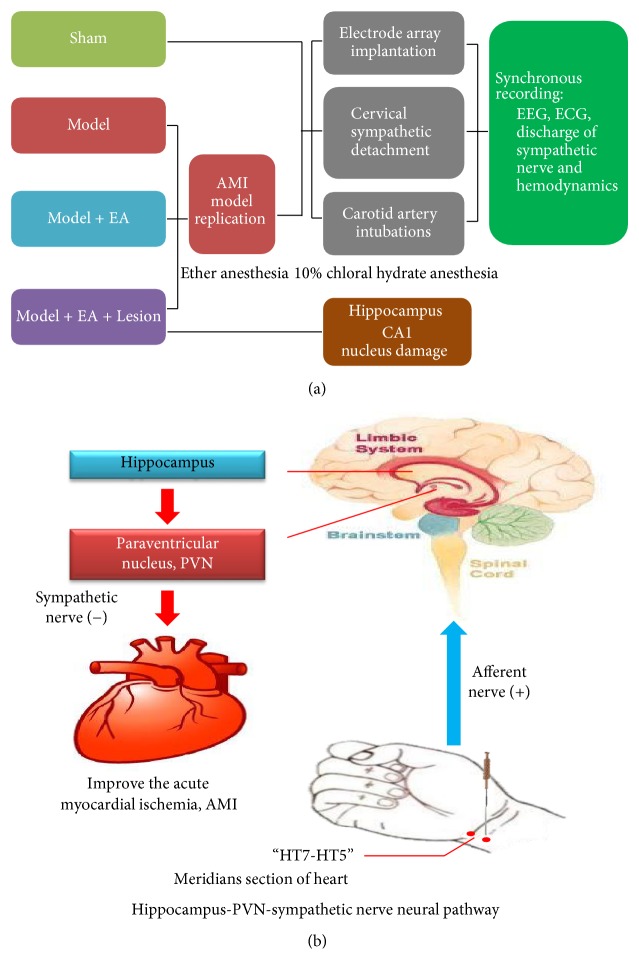
“(a)” showed the flow diagram of experiment. “(b)” showed that the acupuncture signals were sent to the central nervous system via the peripheral nerves, next via the nerve fibers between the hippocampus and PVNs to regulate the excitability of the PVN neurons. Subsequently, the signals were transmitted to the sympathetic nerve via the downstream nerve fibers to regulate cardiac activities, thus achieving the antimyocardial ischemia effect of electroacupuncture.

In summary, together with our previous findings [6], we showed that the number of cells in the hippocampal neuronal discharge was correlated with HR, MAP, and RPP; therefore, we believe that the antimyocardial ischemic effect of electroacupuncture may be achieved via the hippocampus-PVN-sympathetic nerve pathway. After electroacupuncture at the “Shenmen-Tongli” segment, the acupuncture signals were sent to the central nervous system via the peripheral nerves. Next, the signal integration was completed at the hippocampus in the limbic system to regulate the excitability of the PVN neurons via the nerve fibers between the hippocampus and PVNs. Subsequently, the signals were transmitted to the sympathetic nerve via the downstream nerve fibers to regulate cardiac activities, thus achieving the antimyocardial ischemia effect of electroacupuncture (Figure 7(b)).

However, regulation of the cardiovascular system has an inseparable relationship with the nervous system, especially changes in the central nervous system during the development and progression of AMI, which involves the coordinating function of multiple nuclei and multiple brain regions. There is additional ways to achieve regulation of the autonomic nervous system, such as the “hippocampus-NTS-vagus nerve” pathway. Further studies need to be conducted to enrich the overall framework of the cardiovascular domination by the central nervous system to provide a comprehensive and systematic interpretation of the central mechanism of acupuncture for the treatment of myocardial ischemia and to provide a theoretical support for the application of acupuncture in clinical treatment.

## Abbreviations

AMI: Acute myocardial ischemia
EA: Electroacupuncture
PVN: Paraventricular nucleus
MAP: Mean arterial pressure
HR: Heart rate
RPP: Rate pressure product
NTS: Nucleus tractus solitarius
ISI: Interspike interval
LFP: Local field potential.

## Supplementary Materials

Supplementary Materials Figure S1: “A,” “B,” “C,” and “D” are lesion bilateral hippocampal CA1 area; “E” and “F” are the coordinates of PVN. Figure S2: cluster analysis of the discharge frequency of the PVN neurons. “A,” “B,” “C,” and “D” showed that there were 2 PVN neurons with discharge in the Sham group, 1 in the Model group, 4 in the Model + EA group, and 3 in the Model + EA + Lesion group. Figure S3: real-time spectrum analysis of the PVN's LFP discharge. According to the intensity of the spectral energy of the local field potential (LFP), the 4 groups were sequenced as follows: “B” the Model group > “D” the Model + EA + Lesion group > “C” the Model + EA group > “A” the Sham group.
