# Supplementary figures and images for: Electroacupuncture Improved the Function of Myocardial Ischemia Involved in the Hippocampus-Paraventricular Nucleus-Sympathetic Nerve Pathway
Source: Evid Based Complement Alternat Med. 2018 Jan 1;2018:2870676. doi: 10.1155/2018/2870676 (PMC5817851; doi:10.1155/2018/2870676)

## Slide 1
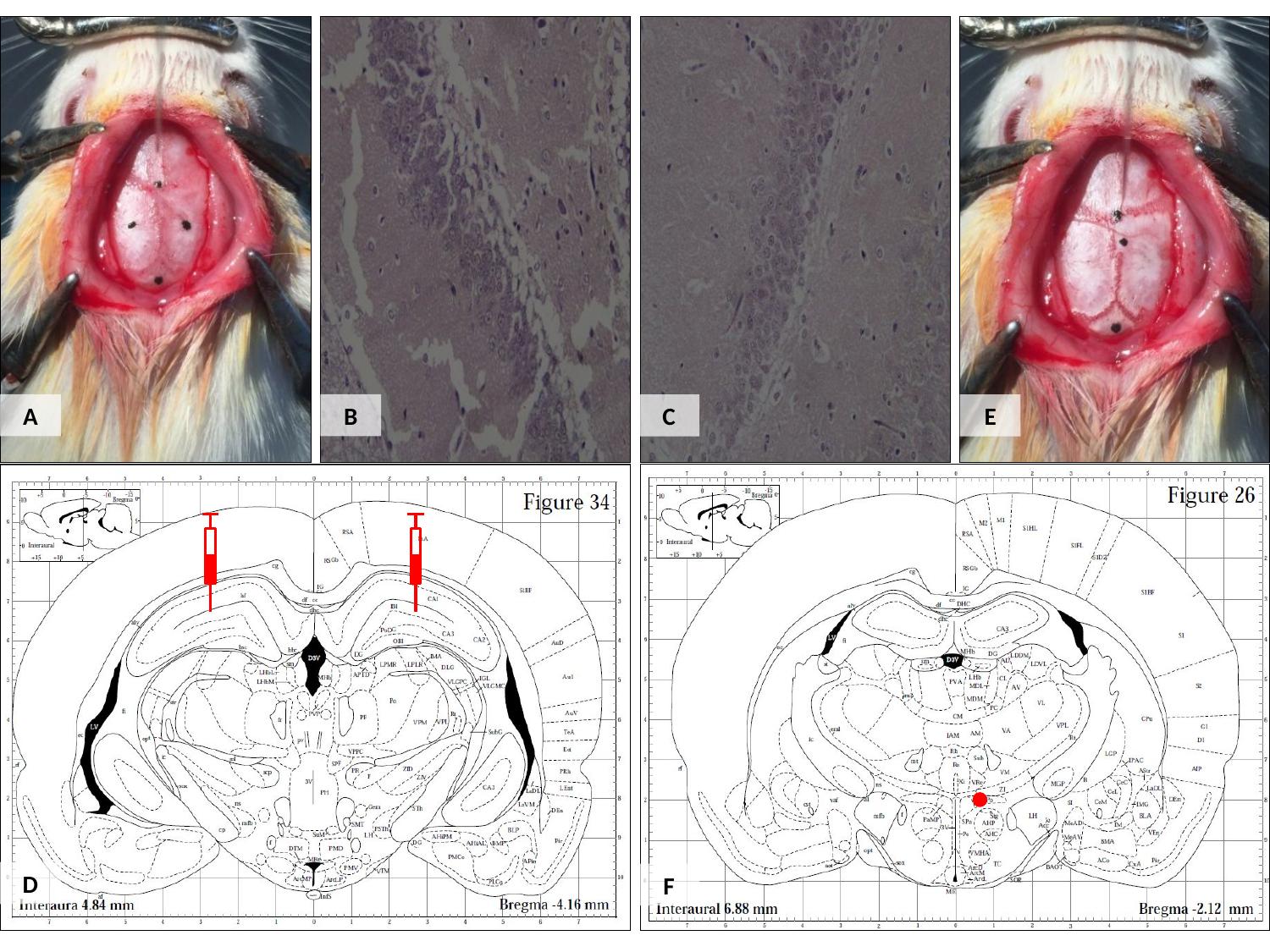

A
B
C
E
D
F

Supplement: Supplementary Materials — Figure S1: “A,” “B,” “C,” and “D” are lesion bilateral hippocampal CA1 area; “E” and “F” are the coordinates of PVN. Figure S2: cluster analysis of the discharge frequency of the PVN neurons. “A,” “B,” “C,” and “D” showed that there were 2 PVN neurons with discharge in the Sham group, 1 in the Model group, 4 in the Model + EA group, and 3 in the Model + EA + Lesion group. Figure S3: real-time spectrum analysis of the PVN's LFP discharge. According to the intensity of the spectral energy of the local field potential (LFP), the 4 groups were sequenced as follows: “B” the Model group > “D” the Model + EA + Lesion group > “C” the Model + EA group > “A” the Sham group. [file 2870676.f1.zip › 2870676.f1/Figure S1_ECAM_2110294.pptx]

## Slide 1
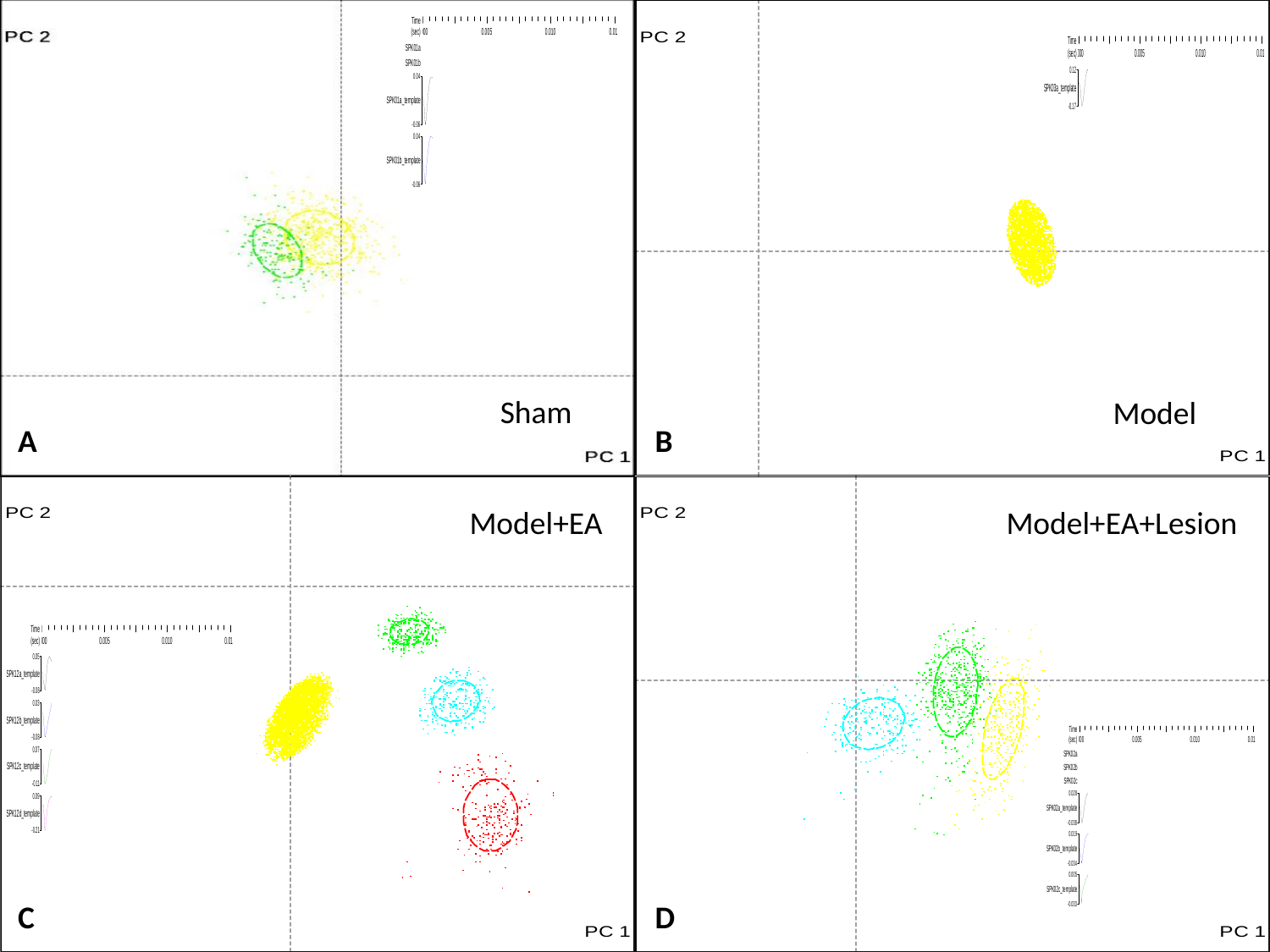

Sham
Model
A
B
Model+EA
Model+EA+Lesion
C
D

Supplement: Supplementary Materials — Figure S1: “A,” “B,” “C,” and “D” are lesion bilateral hippocampal CA1 area; “E” and “F” are the coordinates of PVN. Figure S2: cluster analysis of the discharge frequency of the PVN neurons. “A,” “B,” “C,” and “D” showed that there were 2 PVN neurons with discharge in the Sham group, 1 in the Model group, 4 in the Model + EA group, and 3 in the Model + EA + Lesion group. Figure S3: real-time spectrum analysis of the PVN's LFP discharge. According to the intensity of the spectral energy of the local field potential (LFP), the 4 groups were sequenced as follows: “B” the Model group > “D” the Model + EA + Lesion group > “C” the Model + EA group > “A” the Sham group. [file 2870676.f1.zip › 2870676.f1/Figure S2_ECAM_2110295.pptx]

## Slide 1
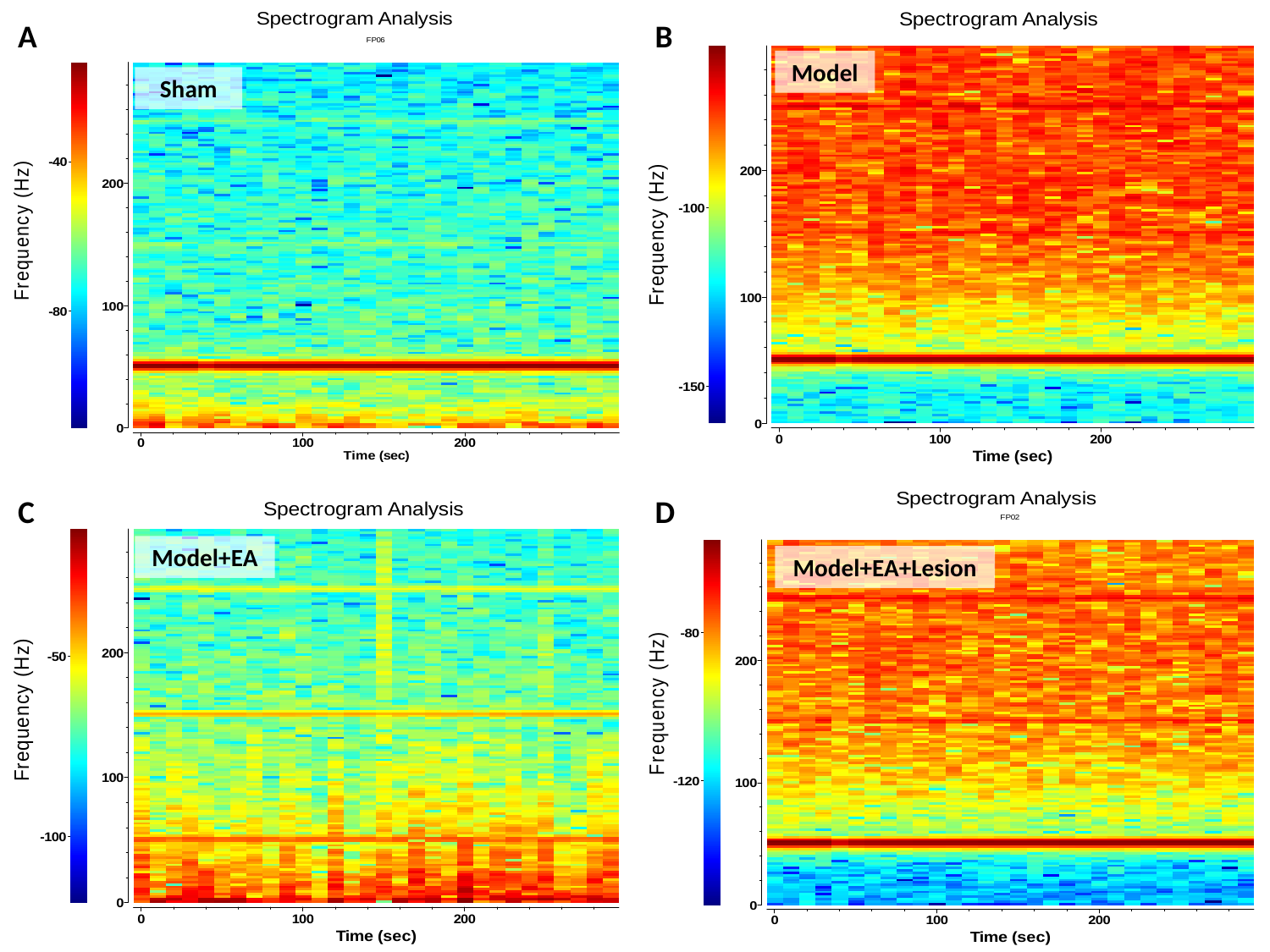

A
B
Model
Sham
C
D
Model+EA
Model+EA+Lesion

Supplement: Supplementary Materials — Figure S1: “A,” “B,” “C,” and “D” are lesion bilateral hippocampal CA1 area; “E” and “F” are the coordinates of PVN. Figure S2: cluster analysis of the discharge frequency of the PVN neurons. “A,” “B,” “C,” and “D” showed that there were 2 PVN neurons with discharge in the Sham group, 1 in the Model group, 4 in the Model + EA group, and 3 in the Model + EA + Lesion group. Figure S3: real-time spectrum analysis of the PVN's LFP discharge. According to the intensity of the spectral energy of the local field potential (LFP), the 4 groups were sequenced as follows: “B” the Model group > “D” the Model + EA + Lesion group > “C” the Model + EA group > “A” the Sham group. [file 2870676.f1.zip › 2870676.f1/Figure S3_ECAM_2110296.pptx]
